# The Active Compounds of Yixin Ningshen Tablet and Their Potential Action Mechanism in Treating Coronary Heart Disease- A Network Pharmacology and Proteomics Approach

**DOI:** 10.1155/2020/4912395

**Published:** 2020-01-25

**Authors:** Xing Lv, Huijun Wang, Ruoming Wu, Xiaoyan Shen, Guan Ye

**Affiliations:** ^1^Central Research Institute, Shanghai Pharmaceuticals Holding Co. Ltd., Shanghai, China; ^2^The MOE Key Laboratory for Standardization of Chinese Medicines and the SATCM Key Laboratory for New Resources and Quality Evaluation of Chinese Medicines, Institute of Chinese Materia Medica, Shanghai University of Traditional Chinese Medicine, Shanghai, China; ^3^Department of Pharmacology, School of Pharmacy, Fudan University, Shanghai, China

## Abstract

Yixin Ningshen tablet is a CFDA-approved TCM formula for treating coronary heart disease (CHD) clinically. However, its active compounds and mechanism of action in treating CHD are unknown. In this study, a novel strategy with the combination of network pharmacology and proteomics was proposed to identify the active components of Yixin Ningshen tablet and the mechanism by which they treat CHD. With the application of network pharmacology, 62 active compounds in Yixin Ningshen tablet were screened out by text mining, and their 313 potential target proteins were identified by a tool in SwissTargetPrediction. These data were integrated with known CHD-related proteomics results to predict the most possible targets, which reduced the 313 potential target proteins to 218. The STRING database was retrieved to find the enriched pathways and related diseases of these target proteins, which indicated that the Calcium, MAPK, PI3K-Akt, cAMP, Rap1, AGE-RAGE, Relaxin, HIF-1, Prolactin, Sphingolipid, Estrogen, IL-17, Jak-STAT signaling pathway, necroptosis, arachidonic acid metabolism, insulin resistance, endocrine resistance, and steroid hormone biosynthesis might be the main pathways regulated by Yixin Ningshen tablet for the treatment of CHD. Through further enrichment analysis and literature study, EGFR, ERBB2, VGFR2, FGF1, ESR1, LOX15, PGH2, HMDH, ADRB1, and ADRB2 were selected and then validated to be the target proteins of Yixin Ningshen tablet by molecular docking, which indicated that Yixin Ningshen tablet might treat CHD mainly through promoting heart regeneration, new vessels' formation, and the blood supply of the myocardial region and reducing cardiac output, oxygen demand, and inflammation as well as arteriosclerosis (promoting vasodilation and intraplaque neoangiogenesis, lowering blood lipid). This study is expected to benefit the clinical application of Yixin Ningshen tablet for the treatment of CHD.

## 1. Introduction

CHD, with clinical condition presented as angina, myocardial infarction, sudden death, and consequent chronic heart failure, is the leading cause of death in noncommunicable diseases and accounted for about 1/3 of all deaths worldwide (17.5 million) in 2012 [1, 2]. Clinical research demonstrated that CHD is an end-stage disease originating from longstanding subclinical atheroma; thus, treatments need to be well designed based on its different stages of pathogenesis [1]. Currently, most researchers have designed new drugs for CHD based on the “one drug for one target for one disease” assumption [3, 4]. For example, lovastatin can slow the progression of coronary atherosclerosis by reducing low-density lipid cholesterol in patients with CHD through targeting 3-hydroxy-3-methylglutaryl-coenzyme A reductase (HMDH). But, because of the complicated etiology and charaterization of CHD, it is hard to treat effectively by intervening at a single node. Then, the combination therapy with multiple drugs is always applied clinically, which could cause adverse off-target effects and adverse drug interactions. Accordingly, safer and more effective treatment strategies for CHD are in urgent need.

Traditional Chinese Medicine (TCM) formulae with low toxicity and high efficiency had been long applied clinically for several thousand years. The mode of action with multicomponent and multitarget is the key feature of TCM formulae. Yixin Ningshen tablet, a CFDA-approved cardiac protective formula, consists of four different herbs: *Schisandra chinensis (Turcz.) Baill.* (*Wu Wei Zi*), *Panax ginseng C. A. Mey* (*Ren Shen*), *Albizzia julibrissin Durazz* (*He Huan Hua*), and *Ganoderma Lucidum Karst* (*Ling Zhi*). In China, its clinical indications include CHD, palpitations, anxiety, depression, neurasthenia, and memory loss. However, until now, no research studies had been conducted to identify its active compounds and potential mechanism of action in treating CHD, which urgently remains to be investigated.

The advent of network pharmacology, whose “multicomponent-multitarget network” theory coincides well with the integrity and systemic nature of TCM formulae, provided us a novel approach for mechanism investigation of TCM formulae [5]. Many previous studies also proved the usefulness of network pharmacology in investigating the synergistic molecular mechanisms of the TCM formula [6–8]. However, false positive probability of network pharmacology approaches is relatively large, which results in hundreds of active components and their potential target proteins for a TCM formula [9–11]. Thus, we try to combine the network pharmacology approach with known proteomics results in order to reduce the false positive probability.

**Figure 1:**
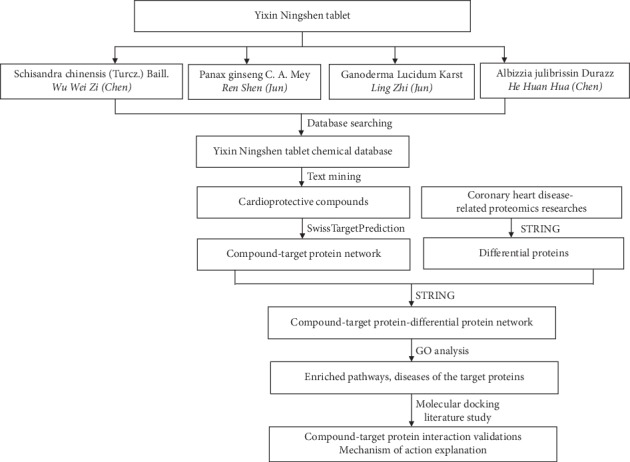
The workflow of the whole study.

In this study, network pharmacology and proteomics were combined to identify the active compounds in Yixin Ningshen tablet and the action mechanisms by which they relieved CHD. The flowchart of this study is shown in Figure 1. This is the first research to study the mechanism of action of Yixin Ningshen tablet for the treatment of CHD.

## 2. Materials and Methods

### 2.1. Database Construction

The compound information (canonical name, structure, and CAS Number) present in Yixin Ningshen tablet was obtained from the TCM Database @ Taiwan (http://tcm.cmu.edu.tw) [12], Traditional Chinese Medicines Integrated Database (http://www.megabionet.org/tcmid) [13], and Chemistry Database (http://www.organchem.csdb.cn). The active compounds of *Wu Wei Zi*, *Ren Shen*, *He Huan Hua*, and *Ling Zhi* were identified by text mining from the National Center of Biotechnology Information PubMed database (http://www.ncbi.nlm.nih.gov/pubmed/). The search bar was composed of a compound name in Yixin Ningshen Tablet and heart or cardiac or myocardial. Compounds which showed cardioprotective effect were regarded as potential active compounds for the treatment of CHD.

### 2.2. Target Prediction of Active Compounds in Yixin Ningshen Tablet

The potential targets of active compounds in Yixin Ningshen tablet were predicted by SwissTargetPrediction [14], which is a website allowing users to predict the targets of a small molecule by the combination of 2D and 3D similarity measures. SwissTargetPrediction compares the query molecule to a library of 280,000 active compounds with more than 2000 targets. In addition, predictions can be made in 5 different organisms, and mapping predictions by homology within and between different species is enabled for close paralogs and orthologs. Extensive cross-validation of the algorithm indicated the reliable performance of SwissTargetPrediction for target prediction, which can be applied in the following experiments. In this study, an active compound-target protein interaction (CTPI) network was constructed and displayed using Cytoscape 3.6.0 [15]. The compounds and the target proteins are linked with edges, and compounds or target proteins are represented by nodes.

### 2.3. CHD-Related Differential Proteins

The CHD-related differential proteins were obtained by text mining from the National Center of Biotechnology Information PubMed database with a search bar (coronary heart disease AND proteomics). Literatures reporting the differential proteins regulated by CHD were downloaded [16–19], and related proteins were summarized.

### 2.4. Network Construction and Analysis

A protein-protein interaction (PPI) network was constructed by uploading the gene names of the potential target proteins and differential proteins regulated by CHD to the public database STRING (version 11.0, https://string-db.org/). The interaction score was set at 0.9 to obtain a high confidence. Cytoscape 3.6.0 was applied to visualize the PPI network. Afterwards, PPI combined with CTPI was used to build a new compound-target protein-differential protein network (CTPP). This network analysis approach can identify target proteins which associate compounds in Yixin Ningshen tablet with differential proteins.

### 2.5. Annotation Enrichment Analysis of Target Proteins

To interpret the biological significance of identified potential targets, GO functional enrichment analysis of STRING database was used to detect their enriched pathway. Terms with false discovery rate <0.05 and observed gene count ≥3 were considered significant.

### 2.6. Molecular Docking

CDOCKER was applied to validate the interation between the active compounds and target proteins. The protein structures were downloaded from RCSB Protein Data Bank (http://www.rcsb.org/) and imported into discovery studio 2.5. Water molecules were then removed from the structures, and the hydrogens were added. The ligand in the cocrystallographic structure was selected and defined as the binding site. At last, the active compounds were imported into discovery studio 2.5 and docked with related proteins using the standard docking protocol.

## 3. Results and Discussion

### 3.1. Active Compounds and Their Potential Target Proteins of Yixin Ningshen Tablet for the Treatment of CHD

Eight hundred and ninety-seven compounds in Yixin Ningshen Tablet were summarized from the TCM databases and references. Also, 62 compounds indicating cardioprotective activity were retained via literature mining, and their structures and other detailed information are shown in Table 1. In addition, a total of 313 potential target proteins (Supplementary Table S1) were obtained through SwissTargetPrediction, of which 130 potential target proteins were enzymes, 39 potential target proteins were membrane receptors, 16 potential target proteins were transcription factors, and 14 potential target proteins were transporters (Supplementary Figure S1). Specifically, there were 34 active compounds in *Ren Shen* targeting 190 potential proteins; 5 active compounds in *Ling Zhi* targeting 56 potential proteins; 18 active compounds in *He Huan Hua* targeting 136 potential proteins, and 9 active compounds in *Wu Wei Zi* targeting 73 potential proteins.

### 3.2. Active Compound-Target Protein Interaction Network Construction and Analysis

**Figure 2:**
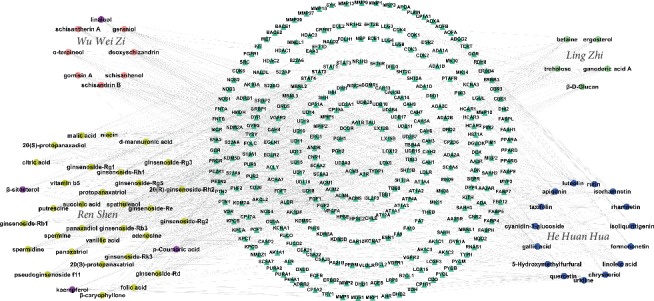
Compound-target protein interaction network of the 62 active compounds from 4 herbs and the potential targets in the treatment of CHD. The olive ellipses are active compounds from *Ren Shen*. The green ellipses represent active compounds of *Ling Zhi*. The pink ellipses represent the active compounds from *Wu Wei Zi*. The blue ellipses are active compounds from *He Huan Hua*. The Purple ellipses represent active compounds in two herbs. The green triangles are the potential targets.

The aforementioned data were used to construct a CPTI (Figure 2), which contains 375 nodes (62 active compounds and 313 potential targets) and 931 edges. In this network, the ellipses and triangles represent the active compounds and their target proteins, respectively. There are some purple ellipses representing the common components of two herbs in Yixin Ningshen tablet. Specifically, *ß*-sitosterol was present in *Ren Shen* and *Wu Wei Zi*; and linalool was an ingredient of both *Wu Wei Zi* and *He Huan Hua*. P-Coumaric acid and kaempferol are constituents of *Ren Shen* and *He Huan Hua*. As shown in Figure 2, there are more active compounds in *Ren Shen* than in *Ling Zhi*, *Wu Wei Zi*, and *He Huan Hua*; thus, the active compounds in *Ren Shen* targeted the largest number of proteins compared with the active compounds in *Ling Zhi*, *Wu Wei Zi*, and *He Huan Hua*. Thus, we infer that *Ren Shen* is the principal ingredient of Yixin Ningshen tablet, which is consistent with the jun-chen-zuo-shi theory in Yixin Ningshen tablet. Interestingly, *Ling Zhi*, same important as *Ren Shen*, has the least number of active compounds in Yixin Ningshen tablet, which may be limited by its number of studies.

### 3.3. Compound-Target Protein-Differential Protein Network Analysis

**Figure 3:**
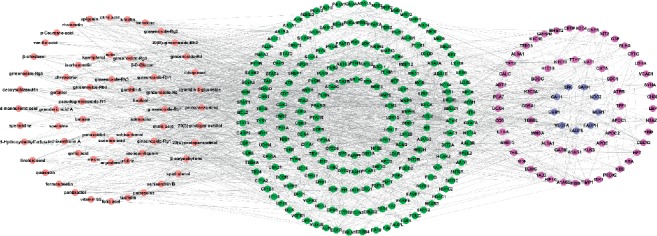
Compound-target protein-differential protein network. This network was constructed by linking 62 active components (pink ellipses) in Yixin Ningshen tablet, 218 target proteins (green and blue ellipses), and 70 differential proteins (purple and blue ellipses).

A compound-target protein-differential protein network (CTPP) was established through the integration of CTPI and PPI to improve the target prediction accuracy of SwissTargetPrediction (Figure 3). This network consists of active compounds, target proteins, and differential proteins, including 341 nodes and 1643 edges. The active compounds targeting related proteins lead to the up- or downregulation of related differential proteins. We searched the STRING database to collect the interaction between target proteins and differential proteins with high confidence (>0.9). The active compounds, target proteins, and differential proteins were regarded as the initial, middle, and terminal nodes, respectively. Figure 3 shows 62 active compounds connecting with 218 target proteins and 70 differential proteins. The 218 target proteins with downstream-regulated differential proteins in this network theoretically have a greater probability to be the true targets of the active compounds in Yixin Ningshen tablet compared with the 313 target proteins identified by SwissTargetPrediction.

### 3.4. Enriched Pathways of the Target Proteins

**Figure 4:**
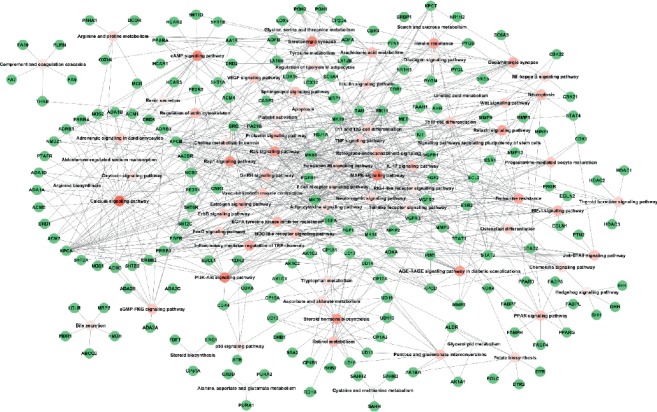
The target protein pathway network. The green ellipses refer to target proteins. The pink ellipses represent pathways, and the deeper the pink, the more the number of target proteins related with pathways.

KEGG pathway analysis was applied to investigate related pathways of the target proteins with the functions in STRING database (Figure 4). The significant pathways that the target proteins are involved in were selected with a *p* value <0.05 and observed gene count ≥3. Generally speaking, pathways with more target proteins involved in are more meaningful than pathways containing fewer target proteins. Thus, a target protein pathway network (Figure 4) was built to depict the importance levels of different pathways. The results indicated that Yixin Ningshen tablet exerted its cardioprotective effects by regulating 78 pathways (Figure 4), of which the calcium signaling pathway, MAPK signaling pathway, PI3K-Akt signaling pathway, cAMP signaling pathway, serotonergic synapse, Rap1 signaling pathway, steroid hormone biosynthesis, Ras signaling pathway, AGE-RAGE signaling pathway in diabetic complications, insulin resistance, relaxin signaling pathway, inflammatory mediator regulation of TRP channels, endocrine resistance, dopaminergic synapse, EGFR tyrosine kinase inhibitor resistance, HIF-1 signaling pathway, necroptosis, arachidonic acid metabolism, retinol metabolism, prolactin signaling pathway, GnRH signaling pathway, sphingolipid signaling pathway, estrogen signaling pathway, regulation of actin cytoskeleton, pentose and glucuronate interconversions, PPAR signaling pathway, ErbB signaling pathway, IL-17 signaling pathway, retrograde endocannabinoid signaling, cGMP-PKG signaling pathway, Jak-STAT signaling pathway, and NOD-like receptor signaling pathway contain 10 target proteins or more. Lots of references demonstrated that CHD was closely related with several pathways, such as calcium signaling pathway (28) [78], MAPK signaling pathway (23) [79], PI3K-Akt signaling pathway (22) [80], cAMP signaling pathway (21) [81], Rap1 signaling pathway (19) [81], steroid hormone biosynthesis (18) [82], AGE-RAGE signaling pathway in diabetic complications (17) [83], insulin resistance (16) [84], relaxin signaling pathway (16) [85], endocrine resistance (15) [86], HIF-1 signaling pathway (12) [87], necroptosis (12) [88], arachidonic acid metabolism (11) [89], prolactin signaling pathway (11) [90], sphingolipid signaling pathway (11) [91], estrogen signaling pathway (11) [92], IL-17 signaling pathway (10) [93], and Jak-STAT signaling pathway (10) [94]. Thus, we infer that Yixin Ningshen tablet may treat CHD primarily by regulating above 18 pathways, which awaits further validation. Of note, pathways with less than 10 target proteins, such as Th17 cell differentiation (9) [95], VEGF signaling pathway (9) [96], Wnt signaling pathway (8) [97], folate biosynthesis (7) [98], tryptophan metabolism (6) [99], adipocytokine signaling pathway (6) [100], toll-like receptor signaling pathway (6) [101], choline metabolism (6) [102], platelet activation (5) [103], and linoleic acid metabolism (3) [104] were also validated to be closely related with CHD, which further proved the reliability of our target identification methods. The active compounds in Yixin Ningshen tablet regulate and restore the network equilibrium by targeting many proteins in many pathways, thereby mitigating the development of CHD. Of note, metabolic pathways and pathways in cancer involving 65 and 42 target proteins, respectively, were not included in Figure 4, the reason was that these two pathways had low selectivity among different diseases.

### 3.5. Construction and Analysis of Target Protein-Disease Network

**Figure 5:**
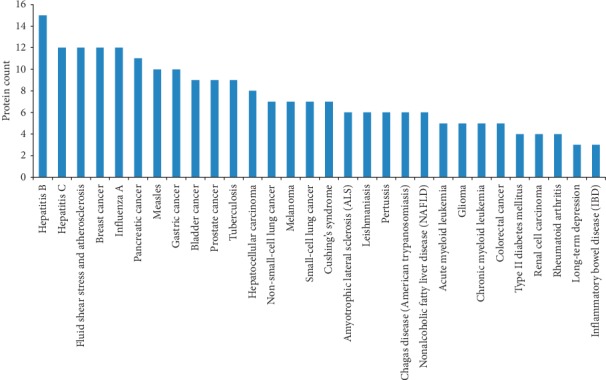
The enriched diseases with more than three potential target proteins by STRING database.

Figure 5 indicates the summarization of the target protein-related diseases and indicates that Yixin Ningshen tablet can show a protective effect against hepatitis B, hepatitis C, fluid shear stress and atherosclerosis, breast cancer, influenza A, pancreatic cancer, measles, gastric cancer, bladder cancer, prostate cancer, tuberculosis, hepatocellular carcinoma, non-small-cell lung cancer, melanoma, small-cell lung cancer, cushing's syndrome, amyotrophic lateral sclerosis (ALS), leishmaniasis, pertussis, chagas disease (American trypanosomiasis), nonalcoholic fatty liver diseases (NAFLD), acute myeloid leukemia, glioma, chronic myeloid leukemia, colorectal cancer, type II diabetes mellitus, renal cell carcinoma, rheumatoid arthritis, long-term depression, and inflammatory bowel disease (IBD). Yixin Ningshen tablet had been applied to treat CHD clinically, and among the aforementioned diseases, fluid shear stress and atherosclerosis had been reported to be a main cause of CHD [105]. Although the combination of CHD-related proteomics results had limited the potential targets predicted by network pharmacology to CHD-related proteins, long-term depression as the other major indication of Yixin Ningshen tablet could still be identified by enrichment analysis of target proteins, which implied that there might be some relationship between CHD and depression.

### 3.6. Target Validation

As SwissTargetPrediction could not provide the interaction details between target proteins and active compounds, here, we applied an accurate docking program called CDOCKER to investigate the binding modes between compounds and target proteins. Aforementioned enrichment analysis indicated the importance of 29 processes, including the calcium signaling pathway, MAPK signaling pathway, PI3K-Akt signaling pathway, cAMP signaling pathway, Rap1 signaling pathway, steroid hormone biosynthesis, AGE-RAGE signaling pathway in diabetic complications, insulin resistance, relaxin signaling pathway, endocrine resistance, HIF-1 signaling pathway, necroptosis, arachidonic acid metabolism, prolactin signaling pathway, sphingolipid signaling pathway, estrogen signaling pathway, IL-17 signaling pathway, Jak-STAT signaling pathway, Th17 cell differentiation, VEGF signaling pathway, Wnt signaling pathway, folate biosynthesis, tryptophan metabolism, adipocytokine signaling pathway, toll-like receptor signaling pathway, choline metabolism, platelet activation, linoleic acid metabolism, and fluid shear stress and atherosclerosis. We selected target proteins related with more than three aforementioned processes and more than three active compounds in Yixin Ningshen tablet, including the epidermal growth factor receptor (EGFR), signal transducer and activator of transcription 3 (STAT3), vascular endothelial growth factor A (VEGFA), signal transducer and activator of transcription 1-alpha/beta (STAT1), receptor tyrosine-protein kinase erbB-2 (ERBB2), vascular endothelial growth factor receptor 2 (VGFR2), muscarinic acetylcholine receptor M1 (ACM1), muscarinic acetylcholine receptor M2 (ACM2), fibroblast growth factor 2 (FGF2), fibroblast growth factor 1 (FGF1), cytochrome P450 1A2 (CP1A2), estrogen receptor (ESR1), arachidonate 15-lipoxygenase (LOX15), cyclin-dependent kinase 4 (CDK4), and prostaglandin G/H synthase 2 (PGH2), as examples to validate. In addition, 3-hydroxy-3-methylglutaryl-coenzyme A reductase (HMDH), *β*1-adrenergic receptor (ADRB1), and β2-adrenergic receptor (ADRB2), as clinically validated target proteins of drugs for CHD, were also selected to validate their interaction with active compounds in Yixin Ningshen tablet. Of note, available protein structures and active pockets were the prerequisites for molecular docking experiments; thus, STAT3, VEGFA, STAT1, FGF2, CP1A2, and CDK4 without known active pockets were excluded from docking validation. A precise docking program called CDOCKER was applied for docking, and the CDOCKER energies were calculated to reflect the binding affinity of compounds with proteins. The binding energies (CDOCKER energies) of compounds with target proteins were listed in Supplementary table S2.

**Figure 6:**
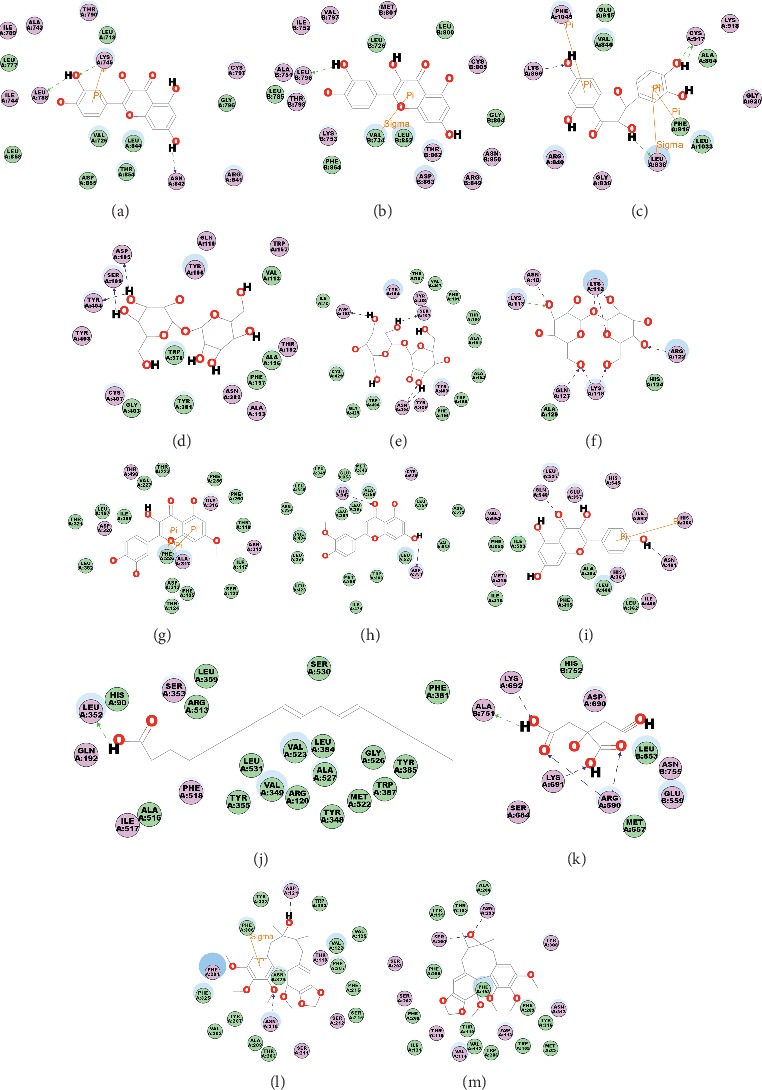
Molecular docking validation results. The binding modes and binding energy of active compounds with their specific potential target proteins were calculated by CDOCKER. (a) The binding form of quercetin and EGFR (PDBID : 2RGP); (b) the binding form of quercetin and ERBB2 (PDBID : 3PP0); (c) the binding mode of taxifolin and VGFR2 (PDBID : 1Y6A); (d) the binding mode of trehalose and ACM1 (PDBID : 5CXV); (e) the binding mode of trehalose and ACM2 (PDBID : 3UON); (f) the binding mode of trehalose and FGF1 (PDBID : 1AFC); (g) the binding mode of chrysoeriol and ESR1 (PDBID : 4XI3); (h) the binding mode of kaempferol and LOX15 (PDBID : 1LOX); (i) the binding mode of linoleic acid and PGH2 (PDBID : 6COX); (j) the binding mode of citric acid and HMDH (PDBID : 1HWJ); (k) the binding mode of gomisin A and ADRB1 (PDBID : 2YCY); and (l) the binding mode of gomisin A and ADRB2 (PDBID : 3NY9).

The interplay between quercetin and EGFR is shown in Figure 6(a), in which the phenolic hydroxy of quercetin could form three hydrogen bonds with Leu788, Lys745, and Asn842; meanwhile, quercetin could also form a *π* cation interaction with Lys745.  Figure 6(b) depicts the binding mode of quercetin and ERBB2. In detail, the phenolic hydroxy of quercetin could form a hydrogen bond with Leu796, and in addition, quercetin had a *π* sigma interaction with Val734.  Figure 6(c) shows that taxifolin binds with VGFR2 by four hydrogen bonds (Lys866, Cys917, and Leu838), two *π*-*π* interactions (Phe916 and Phe1045), and a *π* sigma interaction (Leu838). Trehalose could form three hydrogen bonds with Asp105, Ser109, and Tyr404 in ACM1 (Figure 6(d)). Trehalose could form four hydrogen bonds with Asp103, Ser107, Asn404, and Tyr426 in ACM2 (Figure 6(e)). As to FGF1, trehalose could form nine hydrogen bonds with Asn18, Lys112, Lys113, Lys118, Arg122, and Gln127, respectively (Figure 6(f)). As shown in Figure 6(g), chrysoeriol interacted with ESR1 by two hydrogen bonds (Asp351 and Thr347).  Figure 6(h) indicates that kaempferol binds with LOX15 by two hydrogen bonds (Asn401 and Gln548) and a *π*-*π* interaction (His366).  Figure 6(i) indicates that linoleic acid could form one hydrogen bond with Leu352 in PGH2. The binding mode of citric acid and HMDH is shown in Figure 6(j), in which citric acid could form five hydrogen bonds with Arg590, Lys691, Lys692, and Ala751 in HMDH. The interaction of gomisin A and ADRB1 is presented in Figure 6(k), in which the hydroxy and Oxyl of gomisin A form two hydrogen bonds with Asp121 and Asn310, and gomisin A could also have a *π* sigma interactions with Phe306. The interaction of gomisin A and ADRB2 is presented in Figure 6(l), in which the hydroxy of gomisin A form two hydrogen bonds with Ser204 and Asn293.

Among the aforementioned 12 target proteins, statistical results (Supplementary table S2) showed that *ß*-caryophyllene, protopanaxatriol, kaempferol (also from *He Huan Hua*), citric acid, ginsenoside-Rb1, ginsenoside-Rb3, ginsenoside-Rd, ginsenoside-Re, ginsenoside-Rg1, ginsenoside-Rg3, ginsenoside-Rk3, pseudoginsenoside f11, spathulenol, and *ß*-sitosterol (also from *Wu Wei Zi*) from *Ren Shen* as a principal herb could bind with 2, 2, 2, 1, 1, 1, 1, 1, 1, 1, 1, 1, 1, and 1 target proteins, respectively. In addition, *ß*-D-Glucan, trehalose, and ergosterol from *Ling Zhi* as a principal herb could target 3, 3, and 1 proteins, respectively. Specially, ergosterol was validated to bind with HMDH, a confirmed CHD target protein. Quercetin, formononetin, apigenin, chrysoeriol, isoliquiritigenin, isorhamnetin, linoleic acid, luteolin, rhamnetin, and taxifolin from *He Huan Hua* could bind with 3, 3, 2, 2, 2, 1, 1, 1, 1, and 1 target proteins, respectively. Linalool (also from *He Huan Hua*), *α*-terpineol, gomisin A, schisandrin B, deoxyschizandrin, and schisanhenol from *Wu Wei Zi* could bind with 3, 3, 3, 2, 1 and 1 target proteins, respectively. Aforementioned molecular docking results also validate the interactions obtained from SwissTargetPrediction.

### 3.7. Target Analysis

ACM1 and ACM2, which belonged to muscarinic acetylcholine receptors, had long been regarded as druggable targets for novel therapeutic compounds for the treatment of Alzheimer's disease and other disorders related with impaired cognitive function [106]. However, little references reported their relationships with CHD, which might be attributed to one reason below. The clinical indications of Yixin Ningshen tablet not only include CHD but also include depression, memory loss, and neurasthenia. An inference could be drawn that active compounds in Yixin Ningshen tablet relieved depression and memory loss by targeting ACM1 and ACM2, which remained to be validated further (Figures 6(d) and 6(e)).

We then took aforementioned remained 10 principal validated target proteins as examples to explain the potential synergistic action mechanism of active compounds in Yixin Ningshen tablet for treating CHD. Previous research indicated that transactivation of EGFR had been involved in several cardiovascular conditions, including heart failure, hypertension, and cardiac and vascular hypertrophy [107]. Also, blockade of the EGFR could attenuate vasoconstriction and the progression of hypertension, maintain cardiac function, display a cardiovascular benefit, and could be regarded as a target in cardiovascular disorders. [107] Thereby, quercetin, formononetin, and isoliquiritigenin in *He Huan Hua* might relieve the cardiovascular disorders by antagonizing EGFR.

ERBB2-regualted cardioprotection might be owed to regeneration by increasing the dedifferentiation and proliferation of cardiomyocytes and the induction of hypertrophy and cell survival [108]. Also, experiments also demonstrated that transient reactivation of ERBB2 signaling after myocardial infarction promotes heart regeneration, resulting in cardiomyocyte redifferentiation and tissue replacement with reduced scarring [108]. In addition, research studies also indicated that mice with specific ERBB2 mutation in ventricular cardiomyocytes developed a severe dilated cardiomyopathy [109]. Thus, we inferred that ERBB2 abundant in T-tubules in cardiomyocytes was crucial for adult heart function, and quercetin, formononetin, and isoliquiritigenin in *He Huan Hua* might cure CHD through activating ERBB2 signaling.

VGFR2 was reported to play a unique role in the mediation and promotion of intraplaque neoangiogenesis and thus played a significant role in atherogenesis [110]. Also, a previous study indicated that VGFR2 inhibition could prevent myocardial c-kit+ and CD31+ angiogenesis and reduce inflammation and arteriosclerosis in the allograft [111]. As atherosclerosis had been reported to be a main cause of CHD, we inferred that ginsenoside-Rg1 and ginsenoside-Rk3 in *Ren Shen* and taxifolin in *He Huan Hua* could treat CHD through inhibiting VGFR2.

FGF1 was reported to promote vascular smooth muscle cell hyperplasia in myocardium areas with ischemic injury while direct delivered to the heart via an epicardial sponge [112]. In addition, periadventitial delivery of FGF1 makes the vasomotor regulation normal through endothelium and *ß*-adrenergic-dependent mechanisms and improves myocardial perfusion to the collateral-dependent myocardium, which might be applied to treat patients with severe coronary artery disease [113]. More importantly, gene therapy with FGF1(1–154) could cause angiogenesis in the absence of chronic ischemia. The newly formed collateral blood vessels supplied an anatomical basis for reducing the risk region for myocardial infarction upon the following occlusion of the coronary artery near the site where angiogenesis was induced [114]. A clinical experience also demonstrated that FGF1 application in patients with coronay heart disease could promote the formation of new vessels, increase the blood supply of myocardial region significantly, and become a viable therapeutic approach [115]. Thus, we inferred that ginsenoside-Rb1, ginsenoside-Rb3, ginsenoside-Rd, ginsenoside-Re, ginsenoside-Rg3, and pseudoginsenoside f11 in *Ren Shen*, trehalose, and *ß*-D-Glucan in *Ling Zhi* might cure CHD through binding and activating FGF1 (Figure 6(f)).

In 1997, researchers had reported premature development of coronary atherosclerosis in a man with a disruptive mutation of ESR1 [116]. In addition, ESR1 with a continuous vasodilator and lipid-modulating effect was suggested to be protective against atherosclerosis progression and not trigger clinical episodes as estrogens [117]. Also, selective ESR modulators, such as raloxifene, tamoxifen, and idoxifene, are well known to regulate GPER signaling pathway, which is an important mechanism that inhibits atherosclerosis [116]. Because of the known important role of atherosclerosis in CHD, we inferred that *α*-terpineol and linalool (also in *He Huan Hua*) in *Wu Wei Zi* and apigenin, chrysoeriol, and formononetin in *He Huan Hua* could treat CHD through targeting ESR1(Figure 6(g)).

LOX15 gene disruption in mice could significantly attenuate atherosclerosis [118]. Meanwhile, endothelial-specific over-expression of LOX15 in mice could accelerate the progression of atherosclerosis [119], which indicated the important role of LOX15 in atherosclerosis. Thus, we inferred that kaempferol in *Ren Shen* and *He Huan Hua*, deoxyschizandrin, gomisin A, schisandrin B, and schisanhenol in *Wu Wei Zi* could attenuate CHD by inhibiting LOX15.

A highly selective PGH2 (also named cyclooxygenase-2) inhibitor in LDL receptor-deficient mice was reported to show a protective role in early atherosclerosis [120]. In addition, researchers demonstrated that inhibition of PGH2 with meloxicam could stabilize the atherosclerotic plaque and reduce the recurrence of acute coronary syndromes [121], which further identified PGH2 as a target for treating atherosclerosis. Thus, we inferred that spathulenol in *Ren Shen*, linoleic acid in *He Huan Hua*, and schisandrin B in *Wu Wei Zi* could attenuate CHD by inhibiting PGH2.

HMDH, ADRB1, and ADRB2 are known druggable target proteins for the treatment of CHD. Specifically, the inhibition of HMDH could hinder the process of atherosclerosis and reduce the risk of CHD through limiting the cholesterol biosynthesis, primarily reducing low-density lipoprotein cholesterol. Atorvastatin, fluvastatin, lovastatin, and pravastatin targeting HMDH are indicated as intervention drugs in individuals presenting dyslipidemia at risk of atherosclerotic vascular disease. Metoprolol, atenolol, and bisoprolol targeting ADRB1 and/or ADRB2 are the first-line drugs for the treatment of CHD with antiangina and antiarrhythmia effect. The inhibition of ADRB1 and ADRB2 reduces cardiac output by producing negative chronotropic and inotropic effects without presenting activity towards neither membrane stabilization nor intrinsic sympathomimetics. This reduction in the work demanded of the myocardium also reduces oxygen demand which provides therapeutic benefit by reducing the mismatch of oxygen supply and demand in settings where coronary blood flow is limited, such as in coronary atherosclerosis. Reducing oxygen demand, particularly due to exercise, can reduce the frequency of angina pectoris symptoms. Thus, we inferred that *ß*-sitosterol (also in *Wu Wei Zi)* and citric acid in *Ren Shen* and ergosterol in *Ling Zhi* treated CHD by reducing low-density lipoprotein cholesterol through inhibiting HDMH (Figure 6(h)). In addition, gomisin A in *Ren Shen* could protect against CHD by preventing angina and arrhythmia through antagonizing ADRB1 and ADRB2 (Figures 6(i) and 6(j)).

## 4. Conclusions

A novel strategy with the combination of network pharmacology and proteomics had been proposed and applied to investigate the mechanism of action for the anti-CHD effects of Yixin Ningshen tablet. Text mining was firstly applied to identify 62 components in Yixin Ningshen tablet with cardioprotective activity. Then, 313 potential targets of these compounds were predicted by SwissTargetPrediction. In order to improve the accuracy of target identification, aforementioned network pharmacology results were combined with known proteomics results, which reduced the number of potential target proteins from 313 to 218. Target proteins related pathway enrichment analyses demonstrated that Yixin Ningshen tablet exerted its anti-CHD effect by regulating 29 pathways. Further selective validation demonstrated that Yixin Ningshen tablet might target EGFR, ERBB2, VGFR2, FGF1, ESR1, LOX15, PGH2, HMDH, ADRB1, and ADRB2 and thus promote heart regeneration, new vessels' formation and the blood supply of myocardial region, reduce cardiac output, oxygen demand, inflammation, and arteriosclerosis (promoting vasodilation and intraplaque neoangiogenesis, lowering blood lipid) for the treatment of CHD. Our study provides a new strategy for the study of TCM formulae, and the findings are also expected to provide a basis and reference for the further invesitgation of action mechanism of Yixin Ningshen tablet.

## Figures and Tables

**Table 1:** Active compounds of Yixin Ningshen tablet in treating heart diseases.

No	Compound name	Origin	Formula	Semistructure	Reference
1	d-mannuronic acid	*Ren Shen*	C_6_H_10_O_7_	[H][C@]1(O)O[C@]([H])(C(O)=O)[C@@]([H])(O)[C@]([H])(O)[C@]1([H])O	[20]

2	Spathulenol	*Ren Shen*	C_15_H_24_O	C[C@@]1(CC[C@@H]2[C@@H]1[C@H]3[C@H](C3(C)C)CCC2=C)O	[21]

3	Ginsenoside-rg1	*Ren Shen*	C_42_H_72_O_14_	CC(=CCC[C@@](C)([C@H]1CC[C@@]2([C@@H]1[C@@H](C[C@H]3[C@]2(C[C@@H]([C@@H]4[C@@]3(CC[C@@H](C4(C)C)O)C)O[C@H]5[C@@H]([C@H]([C@@H]([C@H](O5)CO)O)O)O)C)O)C)O[C@H]6[C@@H]([C@H]([C@@H]([C@H](O6)CO)O)O)O)C	[22]

4	Malic acid	*Ren Shen*	C_4_H_6_O_5_	C(C(CC(=O)O)O)(=O)O	[23]

5	Succinic acid	*Ren Shen*	C_4_H_6_O_4_	C(CCC(=O)O)(=O)O	[24]

6	Putrescine	*Ren Shen*	C_4_H_12_N_2_	NCCCCN	[25]

7	Spermidine	*Ren Shen*	C_7_H_19_N_3_	NCCCCNCCCN	[5]

8	Spermine	*Ren Shen*	C_10_H_26_N_4_	C(CCNCCCN)CNCCCN	[26]

9	Panaxatriol	*Ren Shen*	C_30_H_52_O_4_	C[C@@]1(CCCC(O1)(C)C)[C@H]2CC[C@@]3([C@@H]2[C@@H](C[C@H]4[C@]3(C[C@H]([C@@H]5[C@@]4(CC[C@@H](C5(C)C)O)C)O)C)O)C	[27]

10	Panaxadiol	*Ren Shen*	C_30_H_52_O_3_	C[C@@]1(CCCC(O1)(C)C)[C@H]2CC[C@@]3([C@@H]2[C@@H](C[C@H]4[C@]3(CC[C@@H]5[C@@]4(CC[C@@H](C5(C)C)O)C)C)O)C	[28]

11	Pseudoginsenoside f11	*Ren Shen*	C_42_H_72_O_14_	C[C@H]1[C@@H]([C@H]([C@H]([C@@H](O1)O[C@@H]2[C@H]([C@@H]([C@H](O[C@H]2O[C@H]3C[C@@]4([C@H](C[C@H]([C@H]5[C@]4(CC[C@@H]5[C@@]6(CC[C@@H](O6)C(C)(C)O)C)C)O)[C@@]7([C@@H]3C([C@H](CC7)O)(C)C)C)C)CO)O)O)O)O)O	[29]

12	*β*-Caryophyllene	*Ren Shen*	C_15_H_24_	C=C1CC/C=C(C)/CC[C@@]2([H])C(C)(C)C[C@]12[H]	[30]

13	20(S)-Protopanaxatriol	*Ren Shen*	C_30_H_52_O_4_	CC(=CCC[C@@](C)([C@H]1CC[C@@]2([C@@H]1[C@@H](C[C@H]3[C@]2(C[C@@H]([C@@H]4[C@@]3(CC[C@@H](C4(C)C)O)C)O)C)O)C)O)C	[31]

14	Ginsenoside-rd	*Ren Shen*	C_48_H_82_O_18_	CC(=CCC[C@@](C)([C@H]1CC[C@@]2([C@@H]1[C@@H](C[C@H]3[C@]2(CC[C@@H]4[C@@]3(CC[C@@H](C4(C)C)O[C@H]5[C@@H]([C@H]([C@@H]([C@H](O5)CO)O)O)O[C@H]6[C@@H]([C@H]([C@@H]([C@H](O6)CO)O)O)O)C)C)O)C)O[C@H]7[C@@H]([C@H]([C@@H]([C@H](O7)CO)O)O)O)C	[32]

15	Ginsenoside-rk3	*Ren Shen*	C_36_H_60_O_8_	CC(=CCCC(=C)[C@H]1CC[C@@]2([C@@H]1[C@@H](C[C@H]3[C@]2(C[C@@H]([C@@H]4[C@@]3(CC[C@@H](C4(C)C)O)C)O[C@H]5[C@@H]([C@H]([C@@H]([C@H](O5)CO)O)O)O)C)O)C)C	[33]

16	Vanillic acid	*Ren Shen*	C_8_H_8_O_4_	COC1=C(C=CC(=C1)C(=O)O)O	[34]

17	p-Coumaric acid	*Ren Shen; He Huan Hua*	C_9_H_8_O_3_	C1=CC(=CC=C1C=CC(=O)O)O	[35]

18	Kaempferol	*Ren Shen; He Huan Hua*	C_15_H_10_O_6_	O=C(C1=C(O)C=C(O)C=C1O2)C(O)=C2C3=CC=C(O)C=C3	[36]

19	Adenosine	*Ren Shen*	C_10_H_13_N_5_O_4_	C1=NC(=C2C(=N1)N(C=N2)[C@H]3[C@@H]([C@@H]([C@H](O3)CO)O)O)N	[37]

20	Niacin	*Ren Shen*	C_6_H_5_NO_2_	C1=CC(=CN=C1)C(=O)O	[38]

21	20(R)-Ginsenoside-rh2	*Ren Shen*	C_36_H_62_O_8_	CC(=CCC[C@](C)([C@H]1CC[C@@]2([C@@H]1[C@@H](C[C@H]3[C@]2(CC[C@@H]4[C@@]3(CC[C@@H](C4(C)C)O[C@H]5[C@@H]([C@H]([C@@H]([C@H](O5)CO)O)O)O)C)C)O)C)O)C	[39]

22	Ginsenoside-Re	*Ren Shen*	C_48_H_82_O_18_	C[C@H]1[C@@H]([C@H]([C@H]([C@@H](O1)O[C@@H]2[C@H]([C@@H]([C@H](O[C@H]2O[C@H]3C[C@@]4([C@H](C[C@H]([C@H]5[C@]4(CC[C@@H]5[C@](C)(CCC=C(C)C)O[C@H]6[C@@H]([C@H]([C@@H]([C@H](O6)CO)O)O)O)C)O)[C@@]7([C@@H]3C([C@H](CC7)O)(C)C)C)C)CO)O)O)O)O)O	[40]

23	Ginsenoside-rb3	*Ren Shen*	C_53_H_90_O_22_	CC(=CCC[C@@](C)([C@H]1CC[C@@]2([C@@H]1[C@@H](C[C@H]3[C@]2(CC[C@@H]4[C@@]3(CC[C@@H](C4(C)C)O[C@H]5[C@@H]([C@H]([C@@H]([C@H](O5)CO)O)O)O[C@H]6[C@@H]([C@H]([C@@H]([C@H](O6)CO)O)O)O)C)C)O)C)O[C@H]7[C@@H]([C@H]([C@@H]([C@H](O7)CO[C@H]8[C@@H]([C@H]([C@@H](CO8)O)O)O)O)O)O)C	[41]

24	Ginsenoside-rb1	*Ren Shen*	C_54_H_92_O_23_	CC(=CCC[C@@](C)([C@H]1CC[C@@]2([C@@H]1[C@@H](C[C@H]3[C@]2(CC[C@@H]4[C@@]3(CC[C@@H](C4(C)C)O[C@H]5[C@@H]([C@H]([C@@H]([C@H](O5)CO)O)O)O[C@H]6[C@@H]([C@H]([C@@H]([C@H](O6)CO)O)O)O)C)C)O)C)O[C@H]7[C@@H]([C@H]([C@@H]([C@H](O7)CO[C@H]8[C@@H]([C@H]([C@@H]([C@H](O8)CO)O)O)O)O)O)O)C	[42]

25	Ginsenoside-rg3	*Ren Shen*	C_42_H_72_O_13_	CC(=CCCC(C)(C1CCC2(C1C(CC3C2(CCC4C3(CCC(C4(C)C)OC5C(C(C(C(O5)CO)O)O)OC6C(C(C(C(O6)CO)O)O)O)C)C)O)C)O)C	[43]

26	Ginsenoside-rg5	*Ren Shen*	C_42_H_70_O_12_	CC(=CC/C=C(\C)/[C@H]1CC[C@@]2([C@@H]1[C@@H](C[C@H]3[C@]2(CC[C@@H]4[C@@]3(CC[C@@H](C4(C)C)O[C@H]5[C@@H]([C@H]([C@@H]([C@H](O5)CO)O)O)O[C@H]6[C@@H]([C@H]([C@@H]([C@H](O6)CO)O)O)O)C)C)O)C)C	[44]

27	Ginsenoside-rg2	*Ren Shen*	C_42_H_72_O_13_	CC/C(=C/CC[C@@](C)(C1CC[C@]2(C1[C@H](CC3C2C[C@H](C4[C@@]3(CCC(C4(C)C)O)C)O[C@H]5[C@@H]([C@H]([C@@H]([C@H](O5)CO)O)O)O[C@H]6[C@@H]([C@@H]([C@H]([C@@H](O6)C)O)O)O)O)C)O)/C	[45]

28	Ginsenoside-rh1	*Ren Shen*	C_36_H_62_O_9_	CC(=CCCC(C)(C1CCC2(C1C(CC3C2(CC(C4C3(CCC(C4(C)C)O)C)OC5C(C(C(C(O5)CO)O)O)O)C)O)C)O)C	[46]

29	20(S)-Protopanaxadiol	*Ren Shen*	C_30_H_52_O_3_	CC(=CCC[C@@](C)([C@H]1CC[C@@]2([C@@H]1[C@@H](C[C@H]3[C@]2(CC[C@@H]4[C@@]3(CC[C@@H](C4(C)C)O)C)C)O)C)O)C	[47]

30	Protopanaxatriol	*Ren Shen*	C_30_H_52_O_4_	CC(=CCC[C@](C)([C@H]1CC[C@@]2([C@@H]1[C@@H](C[C@H]3[C@]2(C[C@@H]([C@@H]4[C@@]3(CC[C@@H](C4(C)C)O)C)O)C)O)C)O)C	[31]

31	Vitamin b5	*Ren Shen*	C_9_H_17_NO_5_	CC(C)(CO)[C@H](C(=O)NCCC(=O)O)O	[48]

32	Citric acid	*Ren Shen*	C_26_H_29_NO	C(C(=O)O)C(CC(=O)O)(C(=O)O)O	[23]

33	Folic acid	*Ren Shen*	C_19_H_19_N_7_O_6_	C1=CC(=CC=C1C(=O)N[C@@H](CCC(=O)O)C(=O)O)NCC2=CN=C3C(=N2)C(=O)NC(=N3)N	[49]

34	*β*-Sitosterol	*Ren Shen; Wu Wei Zi*	C_29_H_50_O	CC[C@H](CC[C@@H](C)[C@H]1CC[C@@H]2[C@@]1(CC[C@H]3[C@H]2CC=C4[C@@]3(CC[C@@H](C4)O)C)C)C(C)C	[50]

35	*β*-D-Glucan	*Ling Zhi*	C_18_H_32_O_16_	C([C@@H]1[C@H]([C@@H]([C@H]([C@@H](O1)OC2[C@H](O[C@H]([C@@H]([C@H]2O)O)O[C@@H]3[C@H](O[C@@H]([C@@H]([C@H]3O)O)O)CO)CO)O)O)O)O	[51]

36	Ganoderic acid A	*Ling Zhi*	C_30_H_44_O_7_	C[C@H](CC(=O)C[C@@H](C)C(=O)O)[C@H]1C[C@@H]([C@@]2([C@@]1(CC(=O)C3=C2[C@H](C[C@@H]4[C@@]3(CCC(=O)C4(C)C)C)O)C)C)O	[52]

37	Ergosterol	*Ling Zhi*	C_28_H_44_O	C[C@H](/C=C/[C@H](C)C(C)C)[C@H]1CC[C@@H]2[C@@]1(CC[C@H]3C2=CC=C4[C@@]3(CC[C@@H](C4)O)C)C	[53]

38	Betaine	*Ling Zhi*	C_5_H_11_NO_2_	C[N+](C)(C)CC([O-])=O	[54]

39	Trehalose	*Ling Zhi*	C_12_H_22_O_11_	C([C@@H]1[C@H]([C@@H]([C@H]([C@H](O1)O[C@@H]2[C@@H]([C@H]([C@@H]([C@H](O2)CO)O)O)O)O)O)O)O	[55]

40	Cyanidin-3-glucoside	*He Huan Hua*	C_21_H_21_ClO_11_	C1=CC(=C(C=C1C2=[O+]C3=CC(=CC(=C3C=C2O[C@H]4[C@@H]([C@H]([C@@H]([C@H](O4)CO)O)O)O)O)O)O)O.[Cl-]	[56]

41	Gallic acid	*He Huan Hua*	C_7_H_6_O_5_	C1=C(C=C(C(=C1O)O)O)C(=O)O	[57]

42	5-Hydroxymethylfurfural	*He Huan Hua*	C_6_H_6_O_3_	C1=C(OC(=C1)C=O)CO	[58]
43	Quercetin	*He Huan Hua*	C_15_H_10_O_7_	C1=CC(=C(C=C1C2=C(C(=O)C3=C(C=C(C=C3O2)O)O)O)O)O	[59]

44	Uridine	*He Huan Hua*	C_9_H_12_N_2_O_6_	C1=CN(C(=O)NC1=O)[C@H]2[C@@H]([C@@H]([C@H](O2)CO)O)O	[60]

45	Rutin	*He Huan Hua*	C_27_H_30_O_16_	C[C@H]1[C@@H]([C@H]([C@H]([C@@H](O1)OC[C@@H]2[C@H]([C@@H]([C@H]([C@@H](O2)OC3=C(OC4=CC(=CC(=C4C3=O)O)O)C5=CC(=C(C=C5)O)O)O)O)O)O)O)O	[61]

46	Linoleic acid	*He Huan Hua*	C_18_H_32_O_2_	CCCCC/C=C\C/C=C\CCCCCCCC(=O)O	[62]

47	Formononetin	*He Huan Hua*	C_16_H_12_O_4_	COC1=CC=C(C=C1)C2=COC3=C(C2=O)C=CC(=C3)O	[63]

48	Isoliquiritigenin	*He Huan Hua*	C_15_H_12_O_4_	O=C(/C=C/C1=C/C=C(/O)C=C1)C=2C=CC(O)=CC=2O	[64]

49	Rhamnetin	*He Huan Hua*	C_16_H_12_O_7_	O=C2/C1=C(\O)C=C(C=C1O/C(=C2/O)C3=C/C=C(/O)C(O)=C3)OC	[65]

50	Isorhamnetin	*He Huan Hua*	C_16_H_12_O_7_	O=C2/C1=C(\O)C=C(O)C=C1O/C(=C2/O)C3=C/C=C(/O)C(=C3)OC	[66]

51	Chrysoeriol	*He Huan Hua*	C_16_H_12_O_6_	O=C2C=C(OC1=CC(O)=CC(O)=C12)C3=C/C=C(/O)C(=C3)OC	[67]

52	Luteolin	*He Huan Hua*	C_15_H_10_O_6_	O=C2C=C(OC1=CC(O)=CC(O)=C12)C3=C/C=C(/O)C(O)=C3	[68]

53	Apigenin	*He Huan Hua*	C_15_H_10_O_5_	O=C2C=C(OC1=CC(O)=CC(O)=C12)C3=C/C=C(/O)C=C3	[69]

54	Taxifolin	*He Huan Hua*	C_15_H_12_O_7_	C1=CC(=C(C=C1[C@@H]2[C@H](C(=O)C3=C(C=C(C=C3O2)O)O)O)O)O	[70]

55	Schisandrin B	*Wu Wei Zi*	C_23_H_28_O_6_	CC1CC2=CC3=C(OCO3)C(OC)=C2C4=C(OC)C(OC)=C(OC)C=C4CC1C	[71]

56	Schisanhenol	*Wu Wei Zi*	C_23_H_30_O_6_	CC1CC2=CC(OC)=C(OC)C(O)=C2C3=C(OC)C(OC)=C(OC)C=C3CC1C	[72]

57	Deoxyschizandrin	*Wu Wei Zi*	C_24_H_32_O_6_	CC1CC2=CC(=C(C(=C2C3=C(C(=C(C=C3CC1C)OC)OC)OC)OC)OC)OC	[73]

58	Geraniol	*Wu Wei Zi*	C_10_H_18_O	CC(=CCC/C(=C\CO)/C)C	[74]

59	Linalool	*Wu Wei Zi; He Huan Hua*	C_10_H_18_O	CC(=CCCC(C)(C=C)O)C	[75]

60	Schisantherin A	*Wu Wei Zi*	C_30_H_32_O_9_	O[C@]([C@@H](C)C1)(C)[C@@H](OC(C2=CC=CC=C2)=O)C3=CC(OC)=C(OC)C(OC)=C3C4=C1C=C5OCOC5=C4OC	[73]

61	*α*-Terpineol	*Wu Wei Zi*	C_10_H_18_O	OC(C)(C)C1CC=C(C)CC1	[76]

62	Gomisin A	*Wu Wei Zi*	C_23_H_28_O_7_	CC1CC2=CC3=C(C(=C2C4=C(C(=C(C=C4CC1(C)O)OC)OC)OC)OC)OCO3	[77]

## Data Availability

The data used to support the findings of this study are available from the corresponding author upon request.

## Supplementary Materials

Supplementary Materials See Table S1, Table S2, and Figure S1 in the Supplementary Material for comprehensive analysis.
