# Supplementary material for: The Active Compounds of Yixin Ningshen Tablet and Their Potential Action Mechanism in Treating Coronary Heart Disease- A Network Pharmacology and Proteomics Approach
Source: Evid Based Complement Alternat Med. 2020 Jan 25;2020:4912395. doi: 10.1155/2020/4912395 (PMC7204378; doi:10.1155/2020/4912395)
Supplement: Supplementary Materials — See Table S1, Table S2, and Figure S1 in the Supplementary Material for comprehensive analysis. [file 4912395.f1.docx]

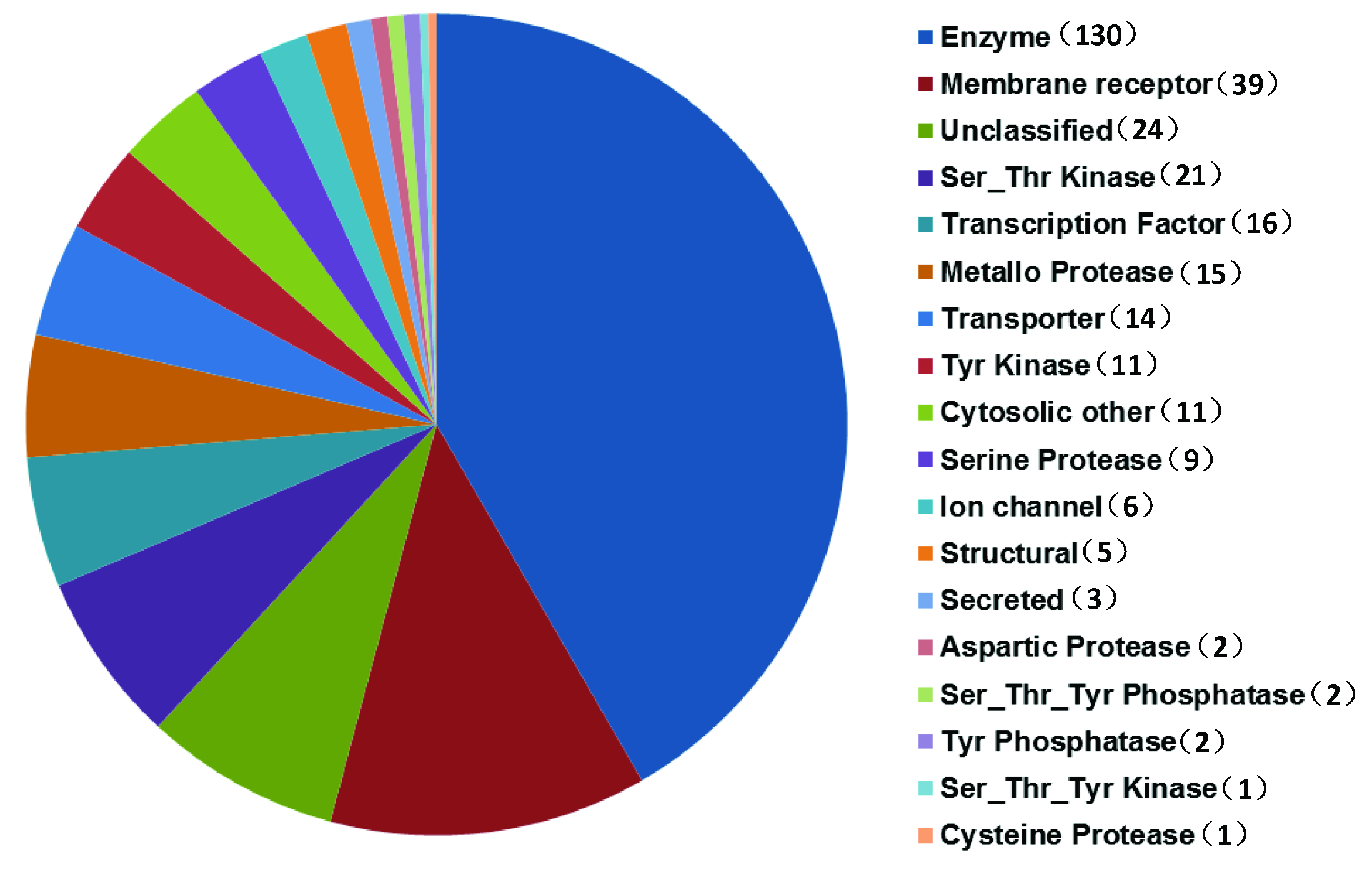


Figure 1: Classification of 316 target proteins identified by SwissTargetPrediction.

Table 1 Potential target proteins of 61 active compounds in Yixin Ningshen Tablet identified by SwissTargetPrediction

| NO | Uniprot ID | Protein name |
| --- | --- | --- |
| 1 | Q13535 | Serine/threonine-protein kinase ATR |
| 2 | Q9NUW8 | Tyrosyl-DNA phosphodiesterase 1 |
| 3 | P09917 | Arachidonate 5-lipoxygenase |
| 4 | P16050 | Arachidonate 15-lipoxygenase |
| 5 | P18054 | Arachidonate 12-lipoxygenase, 12S-type |
| 6 | O15296 | Arachidonate 15-lipoxygenase B |
| 7 | O75342 | Arachidonate 12-lipoxygenase,12R-type |
| 8 | Q9BYJ1 | Epidermis-type lipoxygenase 3 |
| 9 | P10636 | Microtubule-associated protein tau |
| 10 | P30542 | Adenosine receptor A1 |
| 11 | P11926 | Ornithine decarboxylase |
| 12 | Q13627 | Dual specificity tyrosine-phosphorylation-regulated kinase 1A |
| 13 | P23219 | Prostaglandin G/H synthase 1 |
| 14 | P35354 | Prostaglandin G/H synthase 2 |
| 15 | Q9NR56 | Muscleblind-like protein 1 |
| 16 | P21554 | Cannabinoid receptor 1 |
| 17 | P34972 | Cannabinoid receptor 2 |
| 18 | I3L2F9 | Uncharacterized protein |
| 19 | Q9H4B7 | Tubulin beta-1 chain |
| 20 | Q3ZCM7 | Tubulin beta-8 chain |
| 21 | P08908 | 5-hydroxytryptamine receptor 1A |
| 22 | P28222 | 5-hydroxytryptamine receptor 1B |
| 23 | A8K854 | cDNA FLJ16289 fis, clone OCBBF2009920,highly similar to TUBULIN BETA-3 CHAIN |
| 24 | P04350 | Tubulin beta-4A chain |
| 25 | P16662 | UDP-glucuronosyltransferase 2B7 |
| 26 | O75310 | UDP-glucuronosyltransferase 2B11 |
| 27 | O75795 | UDP-glucuronosyltransferase 2B17 |
| 28 | P06133 | UDP-glucuronosyltransferase 2B4 |
| 29 | P36537 | UDP-glucuronosyltransferase 2B10 |
| 30 | P54855 | UDP-glucuronosyltransferase 2B15 |
| 31 | Q6UWM9 | UDP-glucuronosyltransferase 2A3 |
| 32 | Q9BY64 | UDP-glucuronosyltransferase 2B28 |
| 33 | Q9Y4X1 | UDP-glucuronosyltransferase 2A1 |
| 34 | P11511 | Cytochrome P450 19A1 |
| 35 | P30304 | M-phase inducer phosphatase 1 |
| 36 | P30305 | M-phase inducer phosphatase 2 |
| 37 | Q14534 | Squalene monooxygenase |
| 38 | P10275 | Androgen receptor |
| 39 | P23975 | Sodium-dependent noradrenaline transporter |
| 40 | Q01959 | Sodium-dependent dopamine transporter |
| 41 | P11229 | Muscarinic acetylcholine receptor M1 |
| 42 | P08172 | Muscarinic acetylcholine receptor M2 |
| 43 | P08173 | Muscarinic acetylcholine receptor M4 |
| 44 | P08912 | Muscarinic acetylcholine receptor M5 |
| 45 | P20309 | Muscarinic acetylcholine receptor M3 |
| 46 | P22001 | Potassium voltage-gated channel subfamily A member 3 |
| 47 | Q09470 | Potassium voltage-gated channel subfamily A member 1 |
| 48 | Q96RP8 | Potassium voltage-gated channel subfamily A member 7 |
| 49 | P03372 | Estrogen receptor |
| 50 | P00740 | Coagulation factor IXa heavy chain |
| 51 | P00742 | Factor X light chain |
| 52 | P08709 | Coagulation factor VII |
| 53 | P00734 | Activation peptide fragment 1 |
| 54 | Q15759 | Mitogen-activated protein kinase 11 |
| 55 | Q16539 | Mitogen-activated protein kinase 14 |
| 56 | P45983 | Mitogen-activated protein kinase 8 |
| 57 | P45984 | Mitogen-activated protein kinase 9 |
| 58 | P53779 | Mitogen-activated protein kinase 10 |
| 59 | P31645 | Sodium-dependent serotonin transporter |
| 60 | Q92731 | Estrogen receptor beta |
| 61 | P08913 | Alpha-2A adrenergic receptor |
| 62 | P10635 | Cytochrome P450 2D6 |
| 63 | P14416 | D(2) dopamine receptor |
| 64 | P18089 | Alpha-2B adrenergic receptor |
| 65 | P18825 | Alpha-2C adrenergic receptor |
| 66 | P21728 | D(1A) dopamine receptor |
| 67 | P25100 | Alpha-1D adrenergic receptor |
| 68 | P28223 | 5-hydroxytryptamine receptor 2A |
| 69 | P28335 | 5-hydroxytryptamine receptor 2C |
| 70 | P35462 | D(3) dopamine receptor |
| 71 | P41595 | 5-hydroxytryptamine receptor 2B |
| 72 | P50406 | 5-hydroxytryptamine receptor 6 |
| 73 | P28221 | 5-hydroxytryptamine receptor 1D |
| 74 | P55055 | Oxysterols receptor LXR-beta |
| 75 | Q13133 | Oxysterols receptor LXR-alpha |
| 76 | P40763 | Signal transducer and activator of transcription 3 |
| 77 | P42224 | Signal transducer and activator of transcription 1-alpha/beta |
| 78 | P52630 | Signal transducer and activator of transcription 2 |
| 79 | Q14765 | Signal transducer and activator of transcription 4 |
| 80 | Q5VZF2 | Muscleblind-like protein 2 |
| 81 | Q9NUK0 | Muscleblind-like protein 3 |
| 82 | P21918 | D(1B) dopamine receptor |
| 83 | P35968 | Vascular endothelial growth factor receptor 2 |
| 84 | P22748 | Carbonic anhydrase 4 |
| 85 | Q9GZT9 | Egl nine homolog 1 |
| 86 | Q96KS0 | Egl nine homolog 2 |
| 87 | P53396 | ATP-citrate synthase |
| 88 | P36544 | Neuronal acetylcholine receptor subunit alpha-7 |
| 89 | Q494W8 | CHRNA7-FAM7A fusion protein |
| 90 | P37268 | Squalene synthase |
| 91 | O15379 | Histone deacetylase 3 |
| 92 | Q13547 | Histone deacetylase 1 |
| 93 | Q92769 | Histone deacetylase 2 |
| 94 | P15121 | Aldose reductase |
| 95 | C9JRZ8 | Aldo-keto reductase family 1 member B15 |
| 96 | O60218 | Aldo-keto reductase family 1 member B10 |
| 97 | P41229 | Lysine-specific demethylase 5C |
| 98 | Q9UPP1 | Histone lysine demethylase PHF8 |
| 99 | Q9Y2K7 | Lysine-specific demethylase 2A |
| 100 | P29375 | Lysine-specific demethylase 5A |
| 101 | Q9BY66 | Lysine-specific demethylase 5D |
| 102 | Q9UGL1 | Lysine-specific demethylase 5B |
| 103 | O75151 | Lysine-specific demethylase PHF2 |
| 104 | Q6ZMT4 | Lysine-specific demethylase 7 |
| 105 | O43570 | Carbonic anhydrase 12 |
| 106 | P00915 | Carbonic anhydrase 1 |
| 107 | P00918 | Carbonic anhydrase 2 |
| 108 | P07451 | Carbonic anhydrase 3 |
| 109 | P23280 | Carbonic anhydrase 6 |
| 110 | P35218 | Carbonic anhydrase 5A,mitochondrial |
| 111 | P43166 | Carbonic anhydrase 7 |
| 112 | Q16790 | Carbonic anhydrase 9 |
| 113 | Q9ULX7 | Carbonic anhydrase 14 |
| 114 | Q9Y2D0 | Carbonic anhydrase 5B, mitochondrial |
| 115 | Q8N1Q1 | Carbonic anhydrase 13 |
| 116 | Q99720 | Sigma non-opioid intracellular receptor 1 |
| 117 | Q9Y2I1 | Nischarin |
| 118 | P42575 | Caspase-2 subunit p18 |
| 119 | O43323 | Desert hedgehog protein C-product |
| 120 | Q14623 | Indian hedgehog protein N-product |
| 121 | Q15465 | Sonic hedgehog protein C-product |
| 122 | Q16850 | Lanosterol 14-alpha demethylase |
| 123 | P22310 | UDP-glucuronosyltransferase 1-4 |
| 124 | O60656 | UDP-glucuronosyltransferase 1-9 |
| 125 | P19224 | UDP-glucuronosyltransferase 1-6 |
| 126 | P22309 | UDP-glucuronosyltransferase 1-1 |
| 127 | P35503 | UDP-glucuronosyltransferase 1-3 |
| 128 | P35504 | UDP-glucuronosyltransferase 1-5 |
| 129 | Q9HAW7 | UDP-glucuronosyltransferase 1-7 |
| 130 | Q9HAW8 | UDP-glucuronosyltransferase 1-10 |
| 131 | Q9HAW9 | UDP-glucuronosyltransferase 1-8 |
| 132 | P05230 | Fibroblast growth factor 1 |
| 133 | P09038 | Fibroblast growth factor 2 |
| 134 | P15692 | Vascular endothelial growth factor A |
| 135 | Q9Y251 | Heparanase 8 kDa subunit |
| 136 | Q8WWQ2 | Inactive heparanase-2 |
| 137 | P22303 | Acetylcholinesterase |
| 138 | P06276 | Cholinesterase |
| 139 | P54707 | Potassium-transporting ATPase alpha chain 2 |
| 140 | P05023 | Sodium/potassium-transporting ATPase subunit alpha-1 |
| 141 | P13637 | Sodium/potassium-transporting ATPase subunit alpha-3 |
| 142 | P20648 | Potassium-transporting ATPase alpha chain 1 |
| 143 | P50993 | Sodium/potassium-transporting ATPase subunit alpha-2 |
| 144 | Q13733 | Sodium/potassium-transporting ATPase subunit alpha-4 |
| 145 | P01130 | Low-density lipoprotein receptor |
| 146 | P98155 | Very low-density lipoprotein receptor |
| 147 | Q14114 | Low-density lipoprotein receptor-related protein 8 |
| 148 | P10415 | Apoptosis regulator Bcl-2 |
| 149 | Q07817 | Bcl-2-like protein 1 |
| 150 | P17948 | Vascular endothelial growth factor receptor 1 |
| 151 | P35916 | Vascular endothelial growth factor receptor 3 |
| 152 | P42330 | Aldo-keto reductase family 1 member C3 |
| 153 | P52895 | Aldo-keto reductase family 1 member C2 |
| 154 | Q04828 | Aldo-keto reductase family 1 member C1 |
| 155 | P00813 | Adenosine deaminase |
| 156 | P23526 | Adenosylhomocysteinase |
| 157 | P29274 | Adenosine receptor A2a |
| 158 | P29275 | Adenosine receptor A2b |
| 159 | O43865 | Putative adenosylhomocysteinase 2 |
| 160 | Q96HN2 | Putative adenosylhomocysteinase 3 |
| 161 | P04183 | Thymidine kinase,cytosolic |
| 162 | P27487 | Dipeptidyl peptidase 4 membrane form |
| 163 | Q12884 | Seprase |
| 164 | P55263 | Adenosine kinase |
| 165 | P33765 | Adenosine receptor A3 |
| 166 | P27707 | Deoxycytidine kinase |
| 167 | Q16854 | Deoxyguanosine kinase,mitochondrial |
| 168 | P08107 | Heat shock 70 kDa protein 1A/1B |
| 169 | Q8TDS4 | Hydroxycarboxylic acid receptor 2 |
| 170 | P49019 | Hydroxycarboxylic acid receptor 3 |
| 171 | Q9BXC0 | Hydroxycarboxylic acid receptor 1 |
| 172 | P11309 | Serine/threonine-protein kinase pim-1 |
| 173 | Q86V86 | Serine/threonine-protein kinase pim-3 |
| 174 | Q9P1W9 | Serine/threonine-protein kinase pim-2 |
| 175 | P25105 | Platelet-activating factor receptor |
| 176 | P35348 | Alpha-1A adrenergic receptor |
| 177 | P35368 | Alpha-1B adrenergic receptor |
| 178 | P06493 | Cyclin-dependent kinase 1 |
| 179 | P24941 | Cyclin-dependent kinase 2 |
| 180 | P11802 | Cyclin-dependent kinase 4 |
| 181 | Q00526 | Cyclin-dependent kinase 3 |
| 182 | O00214 | Galectin-8 |
| 183 | P17931 | Galectin-3 |
| 184 | P56470 | Galectin-4 |
| 185 | P42892 | Endothelin-converting enzyme 1 |
| 186 | O60344 | Endothelin-converting enzyme 2 |
| 187 | P08473 | Neprilysin |
| 188 | Q495T6 | Membrane metallo-endopeptidase-like 1,soluble form |
| 189 | Q04609 | Glutamate carboxypeptidase 2 |
| 190 | Q9Y3Q0 | N-acetylated-alpha-linked acidic dipeptidase 2 |
| 191 | Q9UQQ1 | N-acetylated-alpha-linked acidic dipeptidase-like protein |
| 192 | P43005 | Excitatory amino acid transporter 3 |
| 193 | P04035 | 3-hydroxy-3-methylglutaryl-coenzyme A reductase |
| 194 | Q4U2R8 | Solute carrier family 22 member 6 |
| 195 | Q63ZE4 | Solute carrier family 22 member 10 |
| 196 | Q6T423 | Solute carrier family 22 member 25 |
| 197 | Q8IVM8 | Solute carrier family 22 member 9 |
| 198 | Q8N4F4 | Solute carrier family 22 member 24 |
| 199 | P05093 | Steroid 17-alpha-hydroxylase/17,20 lyase |
| 200 | Q12772 | Sterol regulatory element-binding protein 2 |
| 201 | P36956 | Sterol regulatory element-binding protein 1 |
| 202 | P00374 | Dihydrofolate reductase |
| 203 | Q86XF0 | Dihydrofolate reductase,mitochondrial |
| 204 | P04818 | Thymidylate synthase |
| 205 | Q05932 | Folylpolyglutamate synthase,mitochondrial |
| 206 | P41440 | Folate transporter 1 |
| 207 | P31939 | IMP cyclohydrolase |
| 208 | P22102 | Phosphoribosylformylglycinamidine cyclo-ligase |
| 209 | Q96NT5 | Proton-coupled folate transporter |
| 210 | P28907 | ADP-ribosyl cyclase 1 |
| 211 | P14550 | Alcohol dehydrogenase [NADP(+)] |
| 212 | Q96JD6 | 1,5-anhydro-D-fructose reductase |
| 213 | Q9NPH5 | NADPH oxidase 4 |
| 214 | P55789 | FAD-linked sulfhydryl oxidase ALR |
| 215 | P14920 | D-amino-acid oxidase |
| 216 | Q99489 | D-aspartate oxidase |
| 217 | P04066 | Tissue alpha-L-fucosidase |
| 218 | Q9BTY2 | Plasma alpha-L-fucosidase |
| 219 | P68400 | Casein kinase II subunit alpha |
| 220 | P19784 | Casein kinase II subunit alpha' |
| 221 | Q8NEV1 | Casein kinase II subunit alpha 3 |
| 222 | P00533 | Epidermal growth factor receptor |
| 223 | P04054 | Phospholipase A2 |
| 224 | P04626 | Receptor tyrosine-protein kinase erbB-2 |
| 225 | P05164 | Myeloperoxidase |
| 226 | P05177 | Cytochrome P450 1A2 |
| 227 | P07477 | Alpha-trypsin chain 1 |
| 228 | P08253 | PEX |
| 229 | P08254 | Stromelysin-1 |
| 230 | P08183 | Multidrug resistance protein 1 |
| 231 | P14061 | Estradiol 17-beta-dehydrogenase 1 |
| 232 | P33527 | Multidrug resistance-associated protein 1 |
| 233 | P35869 | Aryl hydrocarbon receptor |
| 234 | P37059 | Estradiol 17-beta-dehydrogenase 2 |
| 235 | P47989 | Xanthine dehydrogenase/oxidase |
| 236 | Q16678 | Cytochrome P450 1B1 |
| 237 | P32320 | Cytidine deaminase |
| 238 | P06737 | Glycogen phosphorylase,liver form |
| 239 | P11216 | Glycogen phosphorylase,brain form |
| 240 | P11217 | Glycogen phosphorylase,muscle form |
| 241 | P19971 | Thymidine phosphorylase |
| 242 | P07998 | Ribonuclease pancreatic |
| 243 | P16083 | Ribosyldihydronicotinamide dehydrogenase [quinone] |
| 244 | P05413 | Fatty acid-binding protein,heart |
| 245 | P15090 | Fatty acid-binding protein,adipocyte |
| 246 | Q01469 | Fatty acid-binding protein,epidermal |
| 247 | A6NFH5 | Fatty acid-binding protein 12 |
| 248 | O15540 | Fatty acid-binding protein,brain |
| 249 | P02689 | Myelin P2 protein |
| 250 | Q0Z7S8 | Fatty acid-binding protein 9 |
| 251 | P37231 | Peroxisome proliferator-activated receptor gamma |
| 252 | Q03181 | Peroxisome proliferator-activated receptor delta |
| 253 | Q07869 | Peroxisome proliferator-activated receptor alpha |
| 254 | O00519 | Fatty-acid amide hydrolase 1 |
| 255 | P07148 | Fatty acid-binding protein,liver |
| 256 | P80365 | Corticosteroid 11-beta-dehydrogenase isozyme 2 |
| 257 | P16152 | Carbonyl reductase [NADPH] 1 |
| 258 | O75828 | Carbonyl reductase [NADPH] 3 |
| 259 | P21860 | Receptor tyrosine-protein kinase erbB-3 |
| 260 | Q15303 | ERBB4 intracellular domain |
| 261 | Q9NYR8 | Retinol dehydrogenase 8 |
| 262 | Q08257 | Quinone oxidoreductase |
| 263 | O14746 | Telomerase reverse transcriptase |
| 264 | Q9UNQ0 | ATP-binding cassette sub-family G member 2 |
| 265 | P21397 | Amine oxidase [flavin-containing] A |
| 266 | P27338 | Amine oxidase [flavin-containing] B |
| 267 | Q06278 | Aldehyde oxidase |
| 268 | P04798 | Cytochrome P450 1A1 |
| 269 | P00747 | Plasmin light chain B |
| 270 | P08519 | Apolipoprotein(a) |
| 271 | O15438 | Canalicular multispecific organic anion transporter 2 |
| 272 | Q92887 | Canalicular multispecific organic anion transporter 1 |
| 273 | P03956 | 22 kDa interstitial collagenase |
| 274 | P14780 | 67 kDa matrix metalloproteinase-9 |
| 275 | P39900 | Macrophage metalloelastase |
| 276 | P45452 | Collagenase 3 |
| 277 | Q04760 | Lactoylglutathione lyase |
| 278 | Q15046 | Lysine--tRNA ligase |
| 279 | P09238 | Stromelysin-2 |
| 280 | Q9H306 | Matrix metalloproteinase-27 |
| 281 | O60882 | Matrix metalloproteinase-20 |
| 282 | P56817 | Beta-secretase 1 |
| 283 | Q9Y5Z0 | Beta-secretase 2 |
| 284 | P08581 | Hepatocyte growth factor receptor |
| 285 | P10721 | Mast/stem cell growth factor receptor Kit |
| 286 | P11362 | Fibroblast growth factor receptor 1 |
| 287 | P12931 | Proto-oncogene tyrosine-protein kinase Src |
| 288 | Q00534 | Cyclin-dependent kinase 6 |
| 289 | P35228 | Nitric oxide synthase,inducible |
| 290 | P29474 | Nitric oxide synthase,endothelial |
| 291 | P29475 | Nitric oxide synthase,brain |
| 292 | P49354 | Complex |
| 293 | P49356 | Complex |
| 294 | P08235 | Mineralocorticoid receptor |
| 295 | P31213 | 3-oxo-5-alpha-steroid 4-dehydrogenase 2 |
| 296 | P04150 | Glucocorticoid receptor |
| 297 | P06401 | Progesterone receptor |
| 298 | P34995 | Prostaglandin E2 receptor EP1 subtype |
| 299 | P43116 | Prostaglandin E2 receptor EP2 subtype |
| 300 | P17706 | Tyrosine-protein phosphatase non-receptor type 2 |
| 301 | P18031 | Tyrosine-protein phosphatase non-receptor type 1 |
| 302 | P11473 | Vitamin D3 receptor |
| 303 | P05771 | Protein kinase C beta type |
| 304 | P17252 | Protein kinase C alpha type |
| 305 | Q04759 | Protein kinase C theta type |
| 306 | Q05655 | Protein kinase C delta type regulatory subunit |
| 307 | Q05586 | Glutamate receptor ionotropic,NMDA 1 |
| 308 | Q9GZV3 | High affinity choline transporter 1 |
| 309 | P30520 | Adenylosuccinate synthetase isozyme 2 |
| 310 | Q8N142 | Adenylosuccinate synthetase isozyme 1 |
| 311 | P13674 | Prolyl 4-hydroxylase subunit alpha-1 |
| 312 | P07550 | Beta-2 adrenergic receptor |
| 313 | P08588 | Beta-1 adrenergic receptor |

Table 2 The CDOCKER energy between active compounds in Yixin Ningshen tablet and validated target proteins.

| Compound | Target | CDOCKER energy | Compound | Target | CDOCKER energy |
| --- | --- | --- | --- | --- | --- |
| quercetin | EGFR | -39.16 | **rhamnetin** | CP1A2 | -42.5226 |
| positive ligand | EGFR | -31.3827 | **quercetin** | CP1A2 | -39.5432 |
| formononetin | EGFR | -27.5826 | **luteolin** | CP1A2 | -38.4959 |
| isoliquiritigenin | EGFR | -24.3019 | **isorhamnetin** | CP1A2 | -37.2751 |
| positive ligand | ERBB2 | -45.7039 | **kaempferol** | CP1A2 | -36.5577 |
| quercetin | ERBB2 | -35.6649 | **chrysoeriol** | CP1A2 | -36.0736 |
| isoliquiritigenin | ERBB2 | -27.0588 | **apigenin** | CP1A2 | -35.0994 |
| formononetin | ERBB2 | -26.2737 | **positive ligand** | CP1A2 | -27.6543 |
| taxifolin | VGFR2 | -28.2089 | **positive ligand** | ESR1 | -35.8354 |
| positive ligand | VGFR2 | -4.42505 | **chrysoeriol** | ESR1 | -29.913 |
| ginsenoside-Rk3 | VGFR2 | 92.8738 | **apigenin** | ESR1 | -27.8683 |
| ginsenoside-Rg1 | VGFR2 | 103.639 | **formononetin** | ESR1 | -22.5958 |
| positive ligand | ACM2 | -17.7045 | **linalool** | ESR1 | 4.45361 |
| trehalose | ACM2 | -7.21137 | **α-terpineol** | ESR1 | 5.41426 |
| α-terpineol | ACM2 | 0.518294 | **positive ligand** | LOX15 | -30.8301 |
| linalool | ACM2 | 3.34048 | **kaempferol** | LOX15 | -36.0468 |
| β-caryophyllene | ACM2 | 5.50301 | **schisanhenol** | LOX15 | 83.4787 |
| β-D-Glucan | ACM2 | 47.4879 | **schisandrin B** | LOX15 | 118.477 |
| protopanaxatriol | ACM2 | 453.239 | **gomisin A** | LOX15 | 131.345 |
| positive ligand | ACM1 | -28.8668 | **deoxyschizandrin** | LOX15 | 151.03 |
| trehalose | ACM1 | -6.22734 | **positive ligand** | PGH2 | -18.8426 |
| α-terpineol | ACM1 | 0.108233 | **linoleic acid** | PGH2 | -16.84 |
| linalool | ACM1 | 1.57345 | **spathulenol** | PGH2 | 36.046 |
| β-caryophyllene | ACM1 | 15.7837 | **schisandrin B** | PGH2 | 70.863 |
| β-D-Glucan | ACM1 | 41.5559 | **positive ligand** | ADRB1 | -33.1767 |
| protopanaxatriol | ACM1 | 189.234 | **gomisin A** | ADRB1 | 62.2322 |
| trehalose | FGF1 | 4.84144 | **positive ligand** | ADRB2 | -49.4436 |
| β-D-Glucan | FGF1 | 16.3999 | **gomisin A** | ADRB2 | 121.745 |
| positive ligand | FGF1 | 62.936 | **positive ligand** | HMDH | -46.2419 |
| pseudoginsenoside f11 | FGF1 | 102.224 | **citric acid** | HMDH | -36.2664 |
| ginsenoside-Rg3 | FGF1 | 112.017 | **ergosterol** | HMDH | 55.1309 |
| ginsenoside-Rb3 | FGF1 | 127.999 | **β-sitosterol** | HMDH | 32.7252 |
| ginsenoside-Re | FGF1 | 131.681 |  |  |  |
| ginsenoside-Rd | FGF1 | 131.782 |  |  |  |
| ginsenoside-Rb1 | FGF1 | 144.419 |  |  |  |
